# Interobserver Variability in CT Severity Scoring System in COVID-19 Positive Patients

**DOI:** 10.7759/cureus.30193

**Published:** 2022-10-11

**Authors:** Yash Jakhotia, Kajal Mitra, Prashant Onkar, Avinash Dhok

**Affiliations:** 1 Radiodiagnosis, NKP Salve Institute of Medical Sciences and Research Centre (IMSRC) and Lata Mangeshkar Hospital, Nagpur, IND

**Keywords:** reverse transcriptase polymerase chain reaction, ct severity score, computed tomography, pandemic, covid-19

## Abstract

Background: Chest CT scans are done in cases of coronavirus disease 2019 (COVID-19)-positive patients to understand the severity of the disease and plan treatment accordingly. Severity is determined according to a 25-point scoring system, however, there could be interobserver variability in using this scoring system thus leading to the different categorization of patients. We tried to look for this interobserver variability and thus find out its reliability.

Methods: The study was retrospective and was done in a designated COVID center. Some 100 patients were involved in the study who tested positive for COVID-19 disease. The research was conducted over six months (January 2021 to June 2021). Images were given to three radiologists with a minimum of 10 years of experience in thoracic imaging working in different setups at different places for interpretation and scoring further and their scores were compared. Before the study, the local ethics committee granted its approval.

Results: There was no significant variability in the interobserver scoring system thus proving its reliability. The standard deviation between different observers was less than three. There was almost perfect agreement amongst all the observers (Fleiss’ K=0.99 [95% confidence interval, CI: 0.995-0.998]). Maximum variations were observed in the moderate class.

Conclusion: There was minimum inter-observer variability in the 25-point scoring system thus proving its reliability in categorizing patients according to severity. There was no change in the class of the patient according to its severity. A 25-point scoring system hence can be used by clinicians to plan treatment and thus improve a patient's prognosis.

## Introduction

Coronavirus disease 2019 (COVID-19) is a pandemic for the last two years and has spread rapidly all around the world in the last few years and has significantly impacted upon socio-political milieu and healthcare delivery system [1]. It has a wide spectrum of clinical presentations which may vary from asymptomatic patients in form of carriers to patients requiring intensive care [2-3]. At present nasopharyngeal swab test using reverse transcriptase-polymerase chain reaction (RT-PCR) is the gold standard for diagnosing COVID-19 [4]. It is an excellent tool for diagnosing COVID-19, however, has a low sensitivity of 68%, thus missing a significant number of positive COVID-19 patients in form of false negative reports [5].

The CT imaging of the chest plays a significant role and helps in the assessment of patients. Follow-up CTs helps in dynamic evaluation in form of improvement or deterioration of the patient’s condition [6]. Ground glass opacities with or without consolidatory changes are imaging findings in COVID-19-positive patients. The distribution of these ground glass opacities is generally peripheral with bilateral lung involvement [7-8]. Based on imaging findings we could classify patients into different categories in the form of mild, moderate, and severe thus helping treating physicians in their clinical judgment and management of patients [9]. Timely management and proper monitoring of patients requiring intensive care can improve the prognosis in critically ill patients [10-11]. Chest CT with a 25-point visual quantitative evaluation score is used to correlate the severity and thus can be utilized to speed up diagnostic workflow in symptomatic situations [12].

Various studies have been conducted to explore pulmonary involvement using chest CT images with the help of a 25-point visual quantitative assessment; however, our study helps to show inter-observer variability in CT severity grading. Our study compares the CT severity scores of COVID-19-positive patients among three expert radiologists thus helping us in finding out its reliability in the categorization of patients.

Aims and objectives

(*) To find out inter-observer variability in CT severity scoring using a 25-point visual qualitative assessment.

(*) To find out the reliability of CT severity scoring (25-point assessment method).

## Materials and methods

Data collection

The study was conducted after having ethical approval from the college department (NKPSIMS & RC and LMH/IEC/01/2021). From January 2021 to June 2021, chest high resolution computed tomography (HRCT) pictures of COVID-19-positive patients were gathered and analyzed utilizing the picture archiving and communication systems (PACS). The study design was retrospective.

Inclusion criteria

Symptomatic and asymptomatic patients, irrespective of age group and co-morbid conditions, who tested COVID-19 positive by RAT or RT-PCR were included in the study.

Exclusion criteria 

Patients with negative RAT and RT-PCR tests for COVID-19.

Inspection 

The HRCT chest scans of COVID-19-positive patients were evaluated which were done using TOSHIBA 16 slice scanner, Japan. The patient was in the supine position during the scan. Optimum parameters were set according to machine settings. 

Image analysis

Based on CT severity scoring out of 25, three radiologists with a minimum experience of 10 years in thoracic imaging were given images to interpret and score them accordingly. All three radiologists were working in different institutions. Inter-observer survey component was completed after one week and interpretation by the radiologist then was compared.

The scoring system was given as:

According to the percentage involvement of each lobe, a score was given out of five (Table 1). Lung parenchyma was divided into five different lobes with three lobes on the right side and two lobes on the left side (Table 2). Patients were classified into three categories: mild, moderate, and severe based on the scores of the patient (Table 3).

**Table 1:** Scoring system as per percentage involvement.

% Involvement	Score
-	0
<5%	1
5%-25%	2
25%-50%	3
50%-75%	4
>75%	5

**Table 2:** Scoring system as per each lobe involvement. An example of a CT severity scoring system is given where a score has been assigned to each lobe as per the percentage of its involvement.

CT severity	% Involvement	Score
Right upper lobe	-	0
Right middle lobe	<5	1
Right lower lobe	5-25	2
Left upper lobe	25-50	3
Left lower lobe	50-75	4
		Total score: 10/25

**Table 3:** Classification of severity as per scoring system.

Category	Score
Mild	0-8
Moderate	9-15
Severe	16-25

## Results

Descriptive statistics

A comparison of scores was done between three observers and the mean was calculated for each category. The overall mean was 12.23 for the first observer, 12.42 for the second observer, and 12.28 for the third observer (Table 4). The intra-class correlation coefficient was calculated for each of the mild, moderate, and severe categories (Table 5). 

**Table 4:** Comparison of scores between readers. SD, standard deviation

Category of COVID-19	First observer (Mean ± SD)	Second observer (Mean ± SD)	Third observer (Mean ± SD)
Mild	3.31 ± 2.68	3.34 ± 2.80	3.19 ± 2.69
Moderate	11.82 ± 2.00	12.12 ± 2.61	12.00 ± 2.38
Severe	20.77 ± 2.57	21.00 ± 2.93	20.86 ± 2.95
Overall	12.23 ± 7.57	12.42 ± 7.76	12.28 ± 7.73

**Table 5:** Intra-class correlation coefficient. CI, confidence interval Two-way random effects model is used. a. Type C intraclass correlation coefficients using a consistency definition -- the between-measure variance is excluded from the denominator variance. b. The estimator is same, whether the interaction effects is present or not.

Grade	Intraclass co-relation^a^		95% CI		F test with true value 0			
			Lower bound	Upper bound	df1	df2	Sig	
Mild	Single measures	0.958^b^	0.925	0.978	31	62	0.000	
	Average measures	0.985	0.974	0.992	31	62	0.000	
Moderate	Single measures	0.838^b^	0.734	0.910	32	64	0.000	
	Average measures	0.940	0.892	0.968	32	64	0.000	
Severe	Single measures	0.932^b^	0.884	0.962	34	68	0.000	
	Average measures	0.976	0.958	0.987	34	68	0.000	

**Figure 1:**
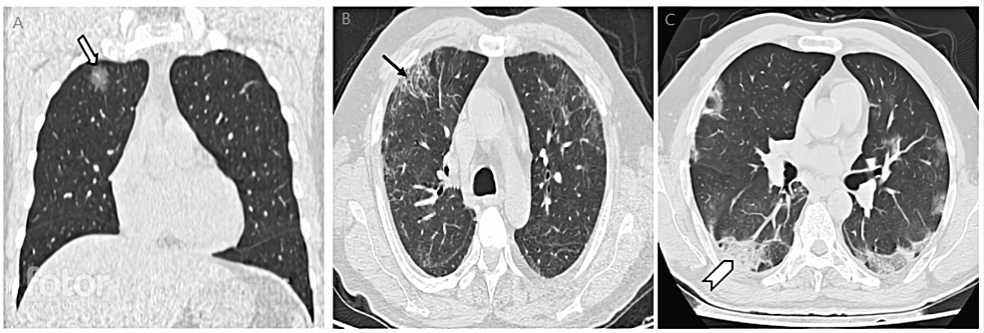
HRCT images of patients classified in mild category. A: Coronal image showing focal GGO in right upper lobe (mild category). Block arrow showing focal ground glass opacity. B: Axial image showing GGOs with adjacent fibrotic changes (mild category). Arrow showing peripheral fibrotic changes. C: Axial image showing GGOs with surrounding consolidatory changes (mild category). Chevron shows peripheral involvement in form of a consolidatory patch. Note focal involvement of lung parenchyma in the mild category. GGO, ground glass opacity; HRCT, high resolution computed tomography

**Figure 2:**
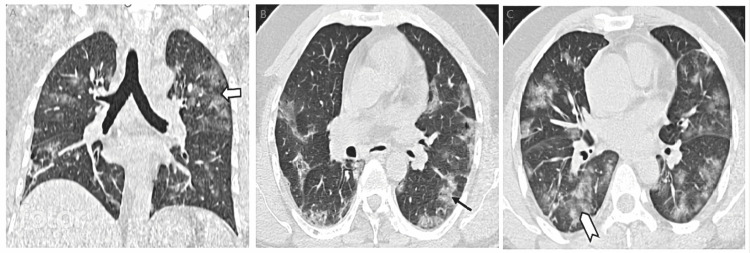
HRCT images of patients classified in moderate category. A: Coronal image showing GGOs involving bilateral lung parenchyma (moderate category). Block arrow showing areas of GGOs. B: Axial image showing GGOs involving bilateral lung parenchyma (moderate category). Arrow showing areas of GGOs with surrounding fibrotic changes. C: Axial image showing GGOs with surrounding consolidatory changes (moderate category). Chevron shows areas of GGOs and consolidatory changes. GGOs, ground glass opacities

**Figure 3:**
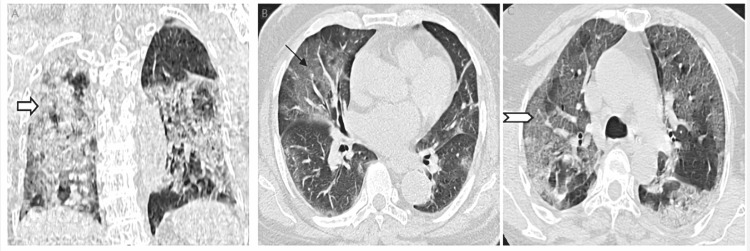
HRCT images of patients classified in severe category. A: Coronal image showing diffuse GGOs involving bilateral lung parenchyma with consolidatory changes (severe category). Block arrow showing diffuse involvement of lung parenchyma with ground glass opacities, consolidatory, and fibrotic changes. B: Axial image showing GGOs involving bilateral lung parenchyma (severe category). Arrow showing diffuse GGO. C: Axial image showing GGOs with surrounding consolidatory changes (severe category). Chevron shows diffuse GGOs and consolidatory changes. Note diffuse involvement of lung parenchyma in the severe category. HRCT, high resolution computed tomography; GGO, ground glass opacity

There was almost perfect agreement among all observers (Fleiss’ K = 0.99 [95% CI: 0.99-0.99]). For various categories of COVID-19, the agreement was excellent in all categories (Table 5). The CT images of patients are classified into different categories as per observers; mild, moderate, and severe (Figures 1-3). Figure 1 shows patients in the mild category, Figure 2 shows patients classified in the moderate category, and Figure 3 shows patients classified in the severe category.

## Discussion

The typical imaging findings of COVID-19 are patchy, rounded, segmental ground glass opacities with or without consolidatory changes. Fibrotic changes along with pleuro-parenchymal bands can be seen in the late stages where fibrosis takes place [13]. HRCT CT is typically not recommended for diagnosing COVID-19. In recent years, the use of chest CT in the management of COVID-19 and the detection of its consequences has grown [14].

Due to its wider availability, COVID-19-positive patients were classified based on HRCT imaging findings in different manners. Classification on the basis of the CO-RADS system was one of the first methods [15]. Patients were also classified according to visually based findings according to the involvement of lung parenchyma into three patterns: peripheral, multifocal, and diffuse findings of pneumonia [16]. Recently patients now are classified according to the 25-point visual quantitative assessment method [9]. It helps in the categorization of patients which further helps in management purposes and determining the prognosis of patients [17]. However, this scoring system is observer-dependent, and thus scores can vary among different radiologists reporting scans. The reliability of this scoring system is still not established and our study helps to find out interobserver variability and thus proves its reliability.

The results in our study regarding interobserver variability in CT severity scoring out of 25 expands the study of Hadied et al. [18] classifying patients according to the Radiological Society of North America (RSNA) classification which states moderate to a substantial agreement, however, in our study there was almost perfect agreement in CT severity scoring system out of 25 and in categorizing patients accordingly.

There is an immense need for a reliable method for reporting CT images of COVID-19-positive patients. For management guidelines, patients who tested positive for COVID-19 were classified into three categories -- mild, moderate, and severe according to CT severity score based on a 25-point assessment method. Based on this classification system, patients are categorized into different categories, which further helps in management purposes. 

However, inter-observer variability if present could play a significant factor, which could change the category of patients and thus affect its management. Our study helps to find out the presence of inter-observer variability if it is present. Variation between inter-observer was not significant as seen by the values from statistics. Maximum variation was seen in the moderate category as compared to mild and severe, however, even that was not significant. There was no change in class of severity between mild, moderate, and severe classes thus proving the reliability of CT severity scoring according to 25-point assessment scoring.

The sample size in this study could be even larger. Additionally, being a retrospective study has its intrinsic limitations such as dependence on previous data and documentation. Also, we did not include cases who were having symptoms yet tested negative for COVID-19 pneumonia.

## Conclusions

It is important for us to categorize patients according to their severity which will help us in knowing patients who require hospital admissions along with clinical evaluation. But to categorize these patients the scoring system needs to be reliable. Results of our study concluded that there was no significant interobserver variability between interobserver in grading the severity of COVID-19 infection out of the 25-point assessment method. In addition, there was no change in class of severity between mild, moderate, and severe categories. Our study proves the reliability of CT severity scoring in grading COVID-19-positive patients according to 25 points, which further helps in deciding management plans for patients.
